# Survival Impacts of Mitochondrial Status in Esophageal Squamous Cell Carcinoma Patients

**DOI:** 10.1245/s10434-024-16533-w

**Published:** 2024-12-07

**Authors:** Kotaro Sugawara, Shingo Sakashita, Takashi Fukuda, Chiaki Murakami, Daiji Oka, Gulanbar Amori, Kumiko Ishibashi, Yasuhito Kobayashi, Hiroaki Kanda, Noriko Motoi

**Affiliations:** 1 Department of Pathology, Saitama Cancer Center, Saitama, Japan; 2 Department of Gastroenterological Surgery, Saitama Cancer Center Hospital, Saitama, Japan; 3 Department of Gastrointestinal Surgery, Graduate School of Medicine, University of Tokyo, Tokyo, Japan; 4 Division of Pathology, Exploratory Oncology Research and Clinical Trial Center, National Cancer Center, Kashiwa, Chiba Japan; 5 Department of Pathology, Saitama Medical Center, Saitama Medical University, Saitama, Japan; 6 Center for Cancer Genomic Medicine, Saitama Cancer Center, Saitama, Japan

**Keywords:** Esophageal squamous cell carcinoma, Neoadjuvant chemotherapy, Mitochondria, Tumor microenvironment

## Abstract

**Background:**

Little is known about the survival impacts of mitochondrial status in esophageal squamous cell carcinoma (ESCC) patients who undergo neoadjuvant chemotherapy (NAC) followed by surgery.

**Methods:**

In total, 260 pre-NAC samples from ESCC patients were analyzed. Mitochondrial status was estimated employing an objective, immunohistochemistry-based system (Mito-score). Mito-scores were dichotomized according to the median value of our cohort. We also evaluated the immune microenvironment (CD4, CD8, Foxp3, HLA class-1, Ki-67 and programmed death ligand-1) on pre-NAC specimens. Multivariate Cox hazards models were applied to determine independent predictors of poor overall survival (OS).

**Results:**

Patients with cT3–4 tumors had higher Mito-scores than those with cT1–2 tumors (*p* = 0.06), and good responders to NAC had significantly higher Mito-scores than poor responders to NAC (*p* = 0.04). CD8 cells and Ki-67 expression were significantly higher in Mito-high than Mito-low tumors (*p* = 0.017 and *p* < 0.001, respectively). Patients with low Mito-scores had significantly poorer OS than those with high Mito-scores (3-year OS: 57.6% vs. 68.2%; *p* = 0.03). A survival difference by Mito-score was evident in cStage III–IV patients (3-year OS: low 50.6% vs. high 66.1%; *p* = 0.006). Multivariable analysis revealed that a low Mito-score (hazard ratio 1.59, 95% confidence interval 1.12–2.24; *p* = 0.009) as well as pT3–4 disease (*p* < 0.001) and pN2–3 disease (*p* < 0.001) were independently associated with poor OS outcomes.

**Conclusions:**

A low Mito-score before NAC had a significant survival impact in ESCC patients, especially in those with advanced disease. Mitochondrial status might be associated with tumor aggressiveness and responsiveness to NAC, thereby possibly affecting the survival outcomes of ESCC patients.

**Supplementary Information:**

The online version contains supplementary material available at 10.1245/s10434-024-16533-w.

Esophageal cancer (EC) is the sixth most common malignancy and the seventh leading cause of cancer death worldwide.^1,2^ The survival outcome of patients with esophageal squamous cell carcinoma (ESCC), especially advanced ESCC, remains unsatisfactory despite the multidisciplinary treatments now available, including surgery, chemotherapy, and radiotherapy.^3^ In East Asia, neoadjuvant chemotherapy (NAC) followed by surgery has now become the standard of care for advanced resectable ESCC.^4,5^

Prior studies have highlighted the crucial role of mitochondria in a wide range of diseases, including neurodegenerative and metabolic disorders,^6^ and have suggested mitochondrial function is closely associated with cellular functions, metabolism, cell growth, and cell death during carcinogenesis.^7,8^ Most cancer cells reportedly rely on aerobic glycolysis, a phenomenon termed ‘the Warburg effect’, which is regarded as reflecting mitochondrial dysfunction or altered metabolism.^9,10^

Evaluating mitochondria in histological specimens of human cancer using immunohistochemistry (IHC) can facilitate improving our understanding of cancer biology. However, IHC evaluation of mitochondria remains difficult because nearly all cells contain mitochondria and their number per cell may have important effects on mitochondrial function. Recently, Sakashita and colleagues proposed an objective mitochondrial evaluation system (Mito-score) for estimating mitochondrial dynamics using machine-based processing of hue, saturation, and value color spaces.^11^

Prior studies raised the possibility that mitochondrial DNA mutations contribute to tumor progression in various malignancies, including lung and gastric cancers.^12–15^ Furthermore, a recent basic research investigation yielded results suggesting that increased mitochondria is associated with the acquisition of gemcitabine resistance in vitro.^16^ Although a few studies have focused on the possible clinical relevance of mitochondrial DNA in ESCC,^17,18^ as well as esophageal adenocarcinoma treated by chemoradiotherapy,^19^ the survival impact of mitochondrial actions has yet to be fully investigated in patients with ESCC.

Herein, using the novel IHC evaluation methodology proposed by Sakashita and colleagues,^11^ we studied the impacts of mitochondrial dynamics in patients with ESCC given NAC followed by surgery.

## Patients and Methods

### Patients

From January 2007 to December 2017, 504 consecutive patients with pathologically confirmed ESCC underwent esophagectomy at the Saitama Cancer Center. Of these 504 patients, 280 who received NAC followed by surgery were eligible for this study. From these 280 patients, we excluded 16 who had synchronous malignancies and 4 whose Mito-score was not obtained from the analysis. Clinical and histological tumor staging was based on the TNM classification (Union for International Cancer Control [UICC], 8th edition).^20^

### Neoadjuvant Therapy and Surgery

NAC followed by surgery was generally performed for patients with clinical Stage (cStage) I, II, III (excluding cT1N0 and cT4b), or IV ESCC due to supraclavicular lymph node (LN) metastasis.^21,22^ During the study period, cisplatin plus 5-fluorouracil (CF) was administered as the standard preoperative therapy, and a regimen consisting of three drugs (cisplatin, 5-fluorouracil, and docetaxel; DCF therapy) was optional. Our standard procedures are subtotal minimally invasive esophagectomy along with en bloc LN dissection using a cervico-thoraco-abdominal approach. The Clavien–Dindo scale was used to grade the severity of all postoperative morbidities.^23^

### Histopathological Evaluation

Pretreatment biopsy specimens were available from all 260 patients. Mitochondrial evaluations were performed using whole tissue sections. Primary tumors were examined, as per the Japanese Classification of Esophageal Cancer, to determine the histological response to preoperative treatment.^24^

### Immunohistochemistry

Immunohistochemical (IHC) staining was performed as previously described.^11^ Briefly, 4-μm-thick sections prepared from formalin-fixed, paraffin-embedded specimens were deparaffinized and rehydrated, and antigen retrieval was then performed. Staining was performed employing rabbit polyclonal anti-cytochrome c oxidase subunit IV isoform 1 (COX4) antibody, using a Roche Ventana BenchMark Ultra autostainer and Ventana Ultra View Universal DAB Detection kit (Roche).

### Immunohistochemical Evaluation

Stained samples were scanned at 40× magnification using a whole slide scanner NanoZoomer S360 (Hamamatsu Photonics, Hamamatsu, Japan). Two investigators (KS and SS, the latter being a certified pathologist) reviewed all specimens and annotated two circled areas of the carcinoma and stroma (0.08 m^2^ each) using Labelme software.^11^ Areas where the carcinoma and stroma showed good separation were selected for the analysis. Spatial annotation of mitochondrial expression and sequential image processing were performed as previously described.^11^ We then extracted the colors yielded by the IHC (brownish coloration produced by DAB) and nuclei (bluish color produced by hematoxylin) by converting an acquired red, green, and blue image to hue, saturation, and value (HSV) color space using the OpenCV library.^11^ IHC evaluation of mitochondria is presented in  Supplemental Figure 1.

### Mito-Score

The Mito-score was defined as the number of COX4 IHC-positive pixels divided by the number of nuclei, which reflects the relationship between the number of mitochondria per cell and their function. The nuclear counting system was applied as previously described.^11^ First, the image threshold of the nucleus was obtained using Otsu’s thresholding technique or discriminant analysis, and a distance map was then created by calculating the distance between the background and the nucleus. Mito-score estimation process  are presented in Supplemental Figures 2 and 3. Certified pathologist (SS) confirmed that the circled area was the nucleus. Mito-scores of the two circled areas were calculated separately and the mean value of these two scores was taken to be the Mito-score of the tumor. Mito-scores were dichotomized according to the median value of our cohort (high- and low-Mito groups).

### Tumor Microenvironment Evaluation

The tumor microenvironment was evaluated as described in our previous reports.^26,27^ The densities of CD4, CD8, Foxp3, HLA class-1, and programmed death-ligand 1 (PD-L1)-positive cells were estimated using HALO software (version 3.4; Indica Labs, Corrales, NM, USA) as previously described. The modified combined positive score (CPS) was also calculated as previously described.^25^ Ki-67-positive cells were analyzed using HALO software (Indica Lab). Specifically, the pathologist removed stroma, manually annotated tumor areas with minimal inflammatory cell infiltration, and simultaneously measured the number of Ki-67-positive cells and the total number of cells in the area, and finally calculated the percentage of positive cells.

### Statistical Analysis

Variables were compared using the Mann–Whitney U test or Chi-square test, as appropriate. Survival curves were constructed using the Kaplan–Meier method, and the log-rank test was used to determine statistical significance, as appropriate. Overall survival (OS) was the period from the date of primary surgery to the date of death from any cause. A multivariate Cox proportional hazards analysis was performed to identify independent prognostic factors. Statistical analyses were carried out using JMP 18.0.0 (SAS Institute, Inc., Cary, NC, USA).

## Results

### Patient Characteristics

Clinicopathological characteristics of our 260 patients are summarized in Table 1. Overall, 75 (29.2%)/165 (64.2%)/17 (6.6%) patients had cStage II/III/IV (due to supraclavicular LN metastasis), respectively. Ninety-three percent of the patients received neoadjuvant CF therapy. The rate of surgery-related deaths was 0.8% (*n* = 2). The distribution of pathological therapeutic effect grade 0–1a/1b/2/3 corresponded to 172 (67.2%)/44 (16.9%)/27 (10.5)/13 (5.0%) patients. Overall, our present series included 35 (13.4)/85 (32.7)/86 (33.1)/54 (20.8) patients with ypStage 0–I/II/III/IV, respectively.

**Table 1:** Characteristics of 260 patients with ESCC

Variables	No. of patients (%)
Age, years [median (range)]	67 (38–81)
Sex, male/female	227 (87.3)/33 (12.7)
Location, Ut-Ce/Mt/Lt-Ae	50 (19.2)/111 (42.7)/99 (38.1)
cStage, II/III/IV	75 (29.2)/165 (64.2)/17 (6.6)
Ki-67 index [median (range)]	34.1 (0–88.1)
Mito-score [median (range)]	19.2 (2–106.1)
NAC regimen	
CF	243 (93.5)
DCF	17 (6.5)
Surgical procedure	
MIE	245 (94.2)
Ivor Lewis	10 (3.8)
Others	5 (2.0)
Postoperative complications	
C-D classification, grade II/III/IV/V	57 (21.9)/53 (20.5)/16 (6.2)/2 (0.8)
Tumor grade	
G1/G2/G3/unknown	19 (7.3)/178 (68.5)/46 (17.7)/17 (6.5)
ypStage	
ypT0–1/T2/T3/T4	89 (34.2)/35 (13.5)/116 (44.6)/20 (7.7)
ypN0/N1/N2/N3	85 (32.7)/96 (36.9)/53 (20.4)/26 (10.0)
ypStage, 0–I/II/III/IV	35 (13.4)/ 85 (32.7)/ 86 (33.1)/ 54 (20.8)
Therapeutic effect, grade 0–1a/1b/2/3/unknown	172 (67.2)/44 (16.9)/27 (10.5)/13 (5.0)/4 (1.4)

*ESCC* esophageal squamous cell carcinoma, *Lt* lower thoracic, *Ae* abdominal, *Mt* middle thoracic, *Ut* upper thoracic, *Ce* cervical, *NAC* neoadjuvant chemotherapy, *CF* cisplatin + 5-fluorouracil, *DCF* docetaxel + cisplatin + 5-fluorouracil, *MIE* minimally invasive esophagectomy, *C-D* Clavien–Dindo

### Mito-Score and Patient Outcomes

The median Mito-score value was 11.35 (range 2.0–106.1, standard deviation 15.8) (Supplementary Fig. 3). We then classified our cohort into two groups according to the median Mito-score value (high-Mito group: ≥ 11.35; low-Mito group: <11.35) and compared patient characteristics between these two groups. The high-Mito group had significantly larger proportions of patients with cT3–4 tumors (56.9% vs. 47.7%; *p* < 0.001) and cStage III–IV disease (77.7% vs. 62.3%; *p* < 0.001) [Table 2]. The good responder (therapeutic effect, grade 2–3) rate was higher in the high-Mito group than in the low-Mito group, but the difference did not reach statistical significance (19.4% vs. 11.8%; *p* = 0.09) [Table 2] (Supplemental Figure 4). Clinicopathological findings were similar in the two groups (Table 2).

**Table 2:** Characteristics of 260 ESCC patients according to Mito-score status

Variables	High Mito-score [*n* = 130]	Low Mito-score[*n* = 130]	*p*-Value
Age, years [median (range)]	68 (38–31)	67 (46–81)	0.92
Sex			0.85
Male	113 (86.9)	114 (87.7)	
Female	17 (13.1)	16 (12.3)	
Location			0.92
Lt-Ae	51 (39.2)	48 (36.9)	
Mt	54 (41.5)	57 (43.9)	
Ut-Ce	25 (19.2)	25 (19.2)	
Tumor grade			0.02
G1	14 (10.8)	5 (3.8)	
G2	87 (66.9)	91 (70.0)	
G3	17 (13.1)	29 (22.3)	
Unknown	12 (9.2)	5 (3.8)	
cStage			
cT3–4	105 (80.8)	79 (60.8)	<0.001
cStage III–IV	101 (77.7)	81 (62.3)	<0.001
ypStage			
ypT3–4	74 (56.9)	62 (47.7)	0.14
ypN0/1/2–3	44 (33.8)/53 (40.8)/33 (25.4)	41 (31.5)/43 (33.1)/46 (35.4)	0.34
ypStage III–IV	70 (53.9)	70 (53.9)	1.00
Lymphovascular invasion	83 (63.9)	94 (72.3)	0.14
Therapeutic effect			
Grade 0–1b/2–3/unknown	104 (80.6)/25 (19.4)/1 (0.8)	112 (88.2)/15 (11.8)/3 (2.3)	0.09

Data are expressed as *n* (%) unless otherwise specified

*ESCC* esophageal squamous cell carcinoma, *Lt* lower thoracic, *Ae* abdominal, *Mt* middle thoracic, *Ut* upper thoracic, *Ce* cervical

Patients with cT3–4 tumors had higher pre Mito-scores than those with cT1–2 tumors (mean 17.93 vs. 13.92; *p* = 0.06) [Supplementary Fig. 4]. The good responder (therapeutic effect, Grade 2–3) group had significantly higher pre Mito-scores than the poor responder (therapeutic effect, Grade 0–1b) group (mean 21.64 vs. 15.97; *p* = 0.04) [Supplementary Fig. 4].

### Survival Outcomes

**Fig. 1:**
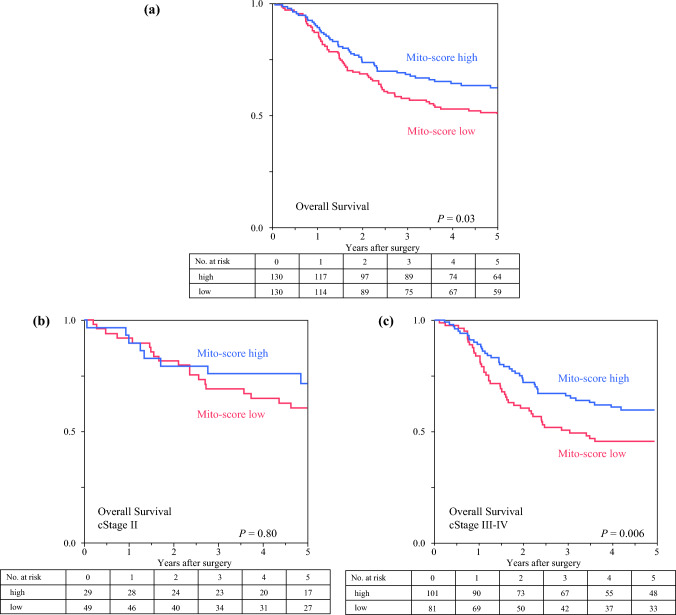
Survival outcomes according to Mito-score. (**a**) Patients with low Mito-scores had significantly poorer overall survival than those with high Mito-scores (*p* = 0.03). (**b**) No significant survival difference was found in cStage II patients (*p* = 0.80), while the (**c**) survival difference was evident in cStage III–IV patients (*p* = 0.006).

We dichotomized Mito-score status as described above and studied the survival impacts of the Mito-score. Patients with a low Mito-score before NAC had significantly poorer OS than those with a high Mito-score before NAC (3-year OS: 57.6% vs. 68.2%; *p* = 0.03) [Fig. 1a]. Subdivision into cStage II and III–IV showed no significant survival difference according to the Mito-score in cStage II patients (3-year OS: low 60.5% vs. high 71.4%; *p* = 0.80) [Fig. 1b], while a survival difference by Mito-score was evident in cStage III–IV patients (3-year OS: low 50.6% vs. high 66.1%; *p* = 0.006) [Fig. 1c].

### Low Mito-Score Before Neoadjuvant Chemotherapy was Shown to be an Independent Predictor for Poor Overall Survival

Univariable analysis and subsequent application of the multivariable Cox proportional hazards model revealed that a low pre Mito-score (hazard ratio [HR] 1.59, 95% confidence interval [CI] 1.12–2.24; *p* = 0.009), as well as pT3–4 disease (HR 2.59, 95% CI 1.79–3.74; *p* < 0.001) and pN2–3 disease (HR 2.19, 95% CI 1.41–3.41; *p* < 0.001), were independently associated with poor OS outcomes (Table 3).

**Table 3:** Cox hazards model for overall survival

Variables	Univariable analysis			Multivariable analysis		
	HR	95% CI	*p*-Value	HR	95% CI	*p*-value
Age >65 years	1.11	0.78–1.57	0.57	1.18	0.83–1.69	0.35
Male	1.53	0.86–2.71	0.15			
Pre Mito-score low (vs. high)	1.44	1.03–2.03	0.03	1.59	1.12–2.24	0.009
cStage III–IV (vs. cStage II)	1.37	0.94–2.01	0.1			
ypT3–4 (vs. pT1–2)	2.76	1.94–3.94	< 0.001	2.59	1.79–3.74	< 0.001
ypN						
ypN0	Ref			Ref		
ypN1	1.42	0.91–2.21	0.12	1.39	0.89–2.17	0.14
ypN2–3	2.9	1.89–4.46	< 0.001	2.19	1.41–3.41	< 0.001

*HR* hazard ratio, *CI* confidence interval, *Ref* reference

### Mito-Score and Tumor Microenvironment

**Fig. 2:**
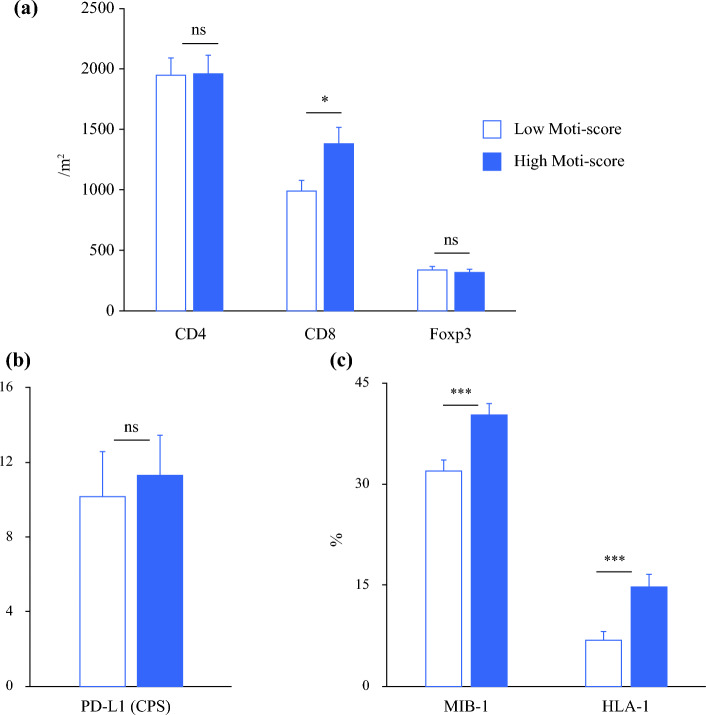
Immune cells according to Mito-scores. The densities of (**a**) CD4 cells (/mm^2^), CD8 cells (/mm^2^) and Foxp3 cells (/mm^2^), (**b**) PD-L1 expression (CPS), and (**c**) MIB-1 cells (%) and HLA-1 cells (%) were determined and were compared according to Mito-scores. The results are presented as the mean ± SEM. A two-tailed Student’s *t* test or the Mann–Whitney nonparametric test was used to determine statistical significance (* *p* < 0.05, ** *p* < 0.01, *** *p* < 0.001). *PD-L1* programmed death-ligand 1, *CPS* combined positive score, *SEM* standard error of the mean, *ns* non-significant

**Fig. 3:**
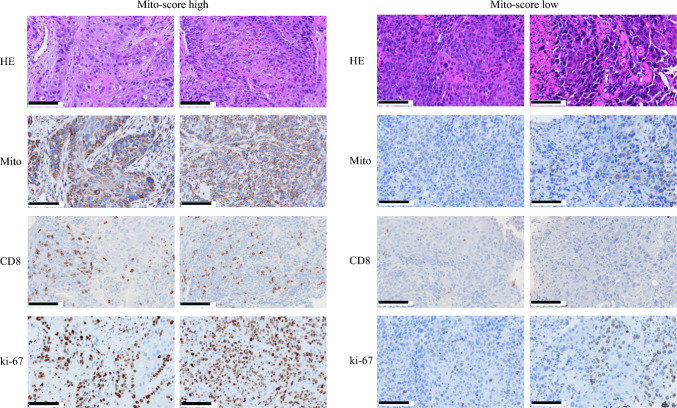
Representative pathological images of high/low Mito-score tumors. Bar represents 100 μm.

Recent studies have suggested mitochondria-related characteristics of tumors were associated with the tumor microenvironment.^29,30^ The densities of CD4 cells were similar in Mito-high and Mito-low tumors (*p* = 0.95) [Fig. 2a], while the density of CD8 cells was significantly higher in the former than in the latter (median 988.2/m^2^ vs. 648.5/m^2^; *p* = 0.017) [Fig. 2a]. PD-L1 expression did not differ significantly between the two groups (*p* = 0.72) [Fig. 2b]. Both Ki-67 and HLA-1 expression were significantly higher in Mito-high than in Mito-low tumors (both *p* < 0.001) [Fig. 2c]. Representative HE and immunohistochemical stained images are presented in Fig. 3.

## Discussion

In this study, we evaluated mitochondrial status in ESCC patients who underwent NAC followed by surgery using a recently proposed, novel mitochondrial evaluation system. Our observations, which suggest that pre-NAC mitochondrial status was associated with NAC response, highlight the independent survival impact of pre-NAC mitochondrial status in the tumor entity.

Previous studies have focused on the possible clinical significance of mitochondria-associated genes.^17,18,26^ A recent study demonstrated that a post-chemoradiotherapy increase in mitochondrial content was associated with treatment resistance in esophageal adenocarcinoma patients.^19^ These studies highlighted the importance of mitochondrial genes in EC patients; however, to our knowledge, no previous studies have examined mitochondrial status, by employing IHC in ESCC patients receiving surgery after NAC, to elucidate the survival impact of mitochondrial status in patients with these tumors.

Visualizing mitochondria in cancer cells from human pathological specimens might enhance our understanding of cancer biology; however, due to the limited methodologies available, relatively few studies have examined mitochondria in human cancer histological specimens using IHC. Sakashita et al. recently established an objective mitochondrial evaluation system using machine-based processing,^11^ which sheds light on the metabolic status of malignant tumors.

We revealed that patients with cT3–4 tumors had higher Mito-scores than those with cT1–2 tumors; however, the mechanisms underlying the association between Mito-score and tumor depth remain to be clarified. Mitochondria are multi-functional; bioenergetics and biosynthetic, while also contributing to cellular stress responses such as autophagy and apoptosis. Both glucose and nitrogen metabolisms are altered during the malignant progression of carcinoma,^27,28^ and they are controlled by nucleotide biosynthesis in the mitochondria.^29,30^ Therefore, Mito-score is expected to be deeply associated with tumor metabolism and tumor progression;^11^ however, further analysis is required to confirm this hypothesis and the biological significance of Mito-score, which quantitatively evaluates mitochondria.

Adenosine triphosphate is mainly synthesized in mitochondria by oxidative phosphorylation, which is regulated by COX, the final and rate-limiting step of the respiratory chain.^31^ We selected an antibody specific for COX4, a marker of the mitochondrial inner membrane, for IHC analysis to evaluate the mitochondria because the granular staining pattern in the cytoplasm clearly indicates the presence of mitochondria. Furthermore, the granular staining pattern of the cytoplasm was better distinguished using COX4 as a marker than voltage-dependent anion-selective channel protein 1, a mitochondrial outer membrane marker.^11^ We defined the Mito-score as the number of COX4 IHC-positive pixels divided by the number of nuclei. This system was validated using various methods in our prior study.^11^ Therefore, this system is applicable to evaluating various carcinomas, including ESCC.

Considering that mitochondria are dynamic organelles and their morphology and other factors change in response to external stimuli and metabolic cues,^32^ we hypothesized that the number of mitochondria recapitulates their function; however, whether number of mitochondria reflects functional status requires further confirmation. Furthermore, we did not analyze the mutation status of mitochondrial DNA in carcinomas or the correlation between the number of mitochondria and the metabolic state. More comprehensive cellular profiling investigations, e.g., RNA-seq or transcriptome profiling, are anticipated to provide more convincing results.

The high-Mito group had significantly higher proportions of patients with cT3–4 tumors and cStage III–IV disease. Furthermore, the high-Mito group showed significantly higher Ki-67 values than the low-Mito group. These results suggest that high-Mito tumors have biological aggressiveness. It is noteworthy that the good responder (therapeutic effect, Grade 2–3) rate was higher in the high-Mito group than in the low-Mito group. Furthermore, the good responder group had a significantly higher pre Mito-score than the poor responder group. Previous studies have revealed that high Ki-67 is associated with good responses to chemotherapy,^33,34^ suggesting that highly proliferative tumors are sensitive to platinum-based chemotherapeutic drugs.^34^ In fact, high Ki-67 levels before chemotherapy were reportedly associated with good response to chemotherapy in breast cancer patients^35,36^ and patients with ESCC.^37^

Furthermore, recent studies have raised the possibility that mitochondrial functions are associated with chemotherapy resistance in gastrointestinal malignancies.^38,39^ Our observations, together with those obtained in these earlier investigations, appear to highlight the potential clinical relevance of mitochondrial status to chemotherapy responsiveness. Overall, our observation that tumors with high pre Mito-score had good responsiveness to NAC is reasonable.

It is noteworthy that in our present study, a low Mito-score was independently associated with poor survival outcomes after adjusting for covariates that might be prognostic factors, suggesting the robust survival impact of a low Mito-score. Mitochondria-associated gene changes are reportedly associated with metabolic pathways, immune activity, and survival in gastrointestinal malignancies.^15,17^ Although glucose and nitrogen metabolism change markedly during the malignant progression of carcinomas, inducing the expressions of various enzymes and leading to mitochondrial dysfunctions,^27,29^ the precise mechanisms underlying the independent survival impact of a low Mito-score have yet to be elucidated.

Limitations must be taken into account when interpreting the results of this study. First, as mentioned above, a precise evaluation methodology for mitochondria remains to be established and validated for various specific malignancies, including ESCC. Unlike methods based on deep learning, our approach allows for parameter adjustments to tailor the conditions to each facility, suggesting the potential for tuning to suit various settings;^11^ however, the adaptability of our method has yet to be fully explored. Second, our cohort comprised only patients who underwent NAC followed by surgery, and we evaluated only specimens collected before NAC. Mitochondrial dynamics change markedly after chemotherapy, which reportedly contributes to resistance to chemotherapy in tumors of the gastrointestinal tract.^16,19^ Third, intratumoral heterogeneity might have affected our results. Although we calculated Mito-scores of two areas in each tumor, in an effort to minimize the influence of intratumoral heterogeneity, evaluation of the tumor microenvironment in small tumors, such as early-stage resections or biopsy samples, are readily affected by intratumoral heterogeneity.^40,41^ While a recent investigation showed substantial spatial heterogeneity between primary and metastatic tumors,^42^ we did not evaluate the spatial heterogeneity of the tumors in this study. Fourth, we employed the median value of Mito-score in our cohort as the threshold, although the rationale for the Mito-score threshold is not fully addressed in this study, possibly limiting the application of Mito-score in the clinical setting.

## Conclusion

A low Mito-score before NAC was shown to be an independent predictor of poor survival outcomes in ESCC patients who underwent NAC followed by surgery. The survival impact of mitochondrial status was evident in patients with advanced disease. Mitochondrial status before NAC might be associated with tumor aggressiveness and the response to chemotherapy, thereby possibly impacting the survival outcomes of patients with ESCC.

## Supplementary Information

Below is the link to the electronic supplementary material.

Supplementary file2 (TIFF 20401 kb)

Supplementary file3 (TIFF 17855 kb)

Supplementary file4 (TIFF 11588 kb)

Supplementary file5 (TIFF 4675 kb)
