# Supplementary figures and images for: Survival Impacts of Mitochondrial Status in Esophageal Squamous Cell Carcinoma Patients
Source: Ann Surg Oncol. 2024 Dec 7;32(3):1963–72. doi: 10.1245/s10434-024-16533-w (PMC11811432; doi:10.1245/s10434-024-16533-w)

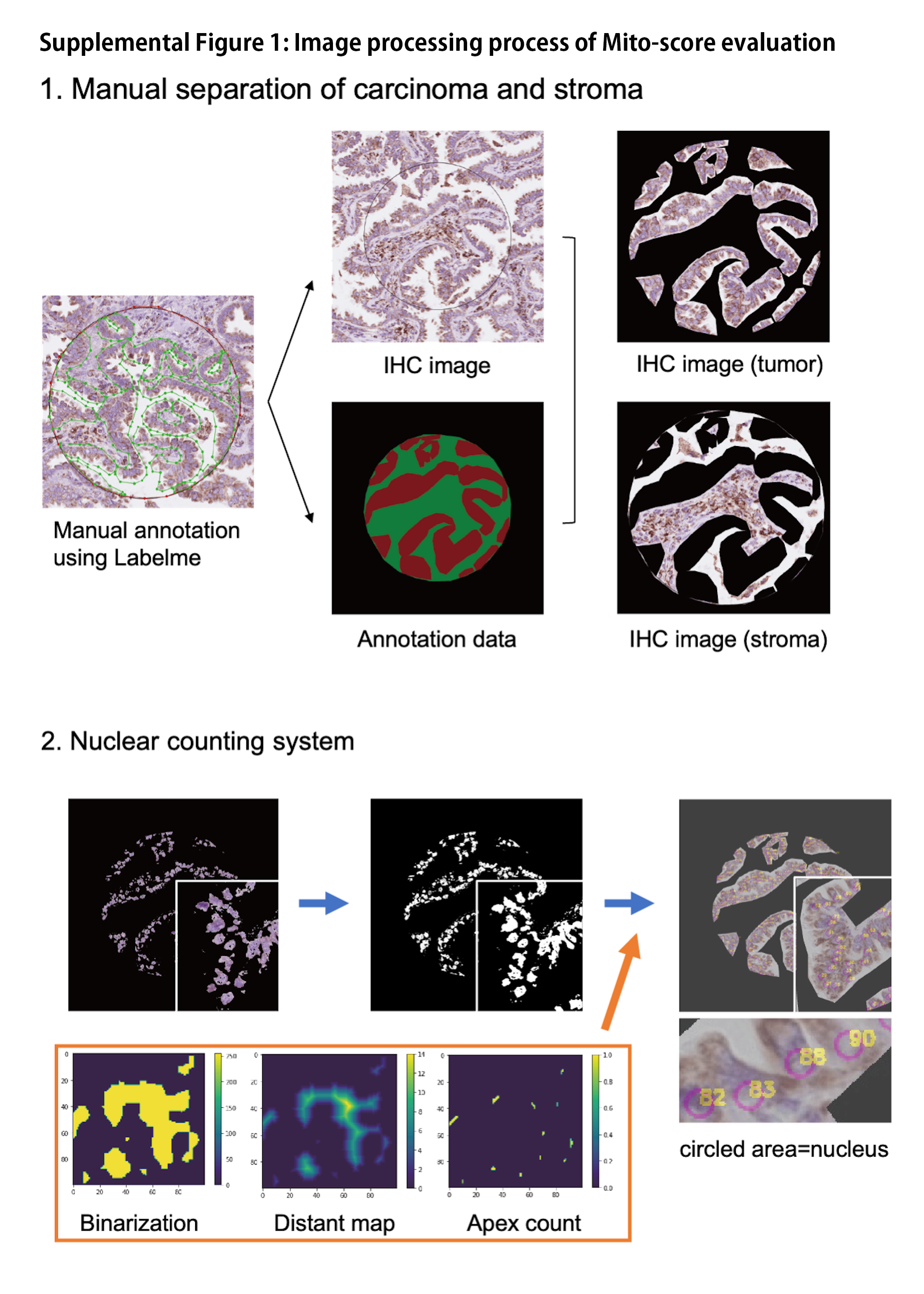

Supplement: Supplementary file 1 — Supplementary file2 (TIFF 20401 kb) [file 10434_2024_16533_MOESM1_ESM.tif]

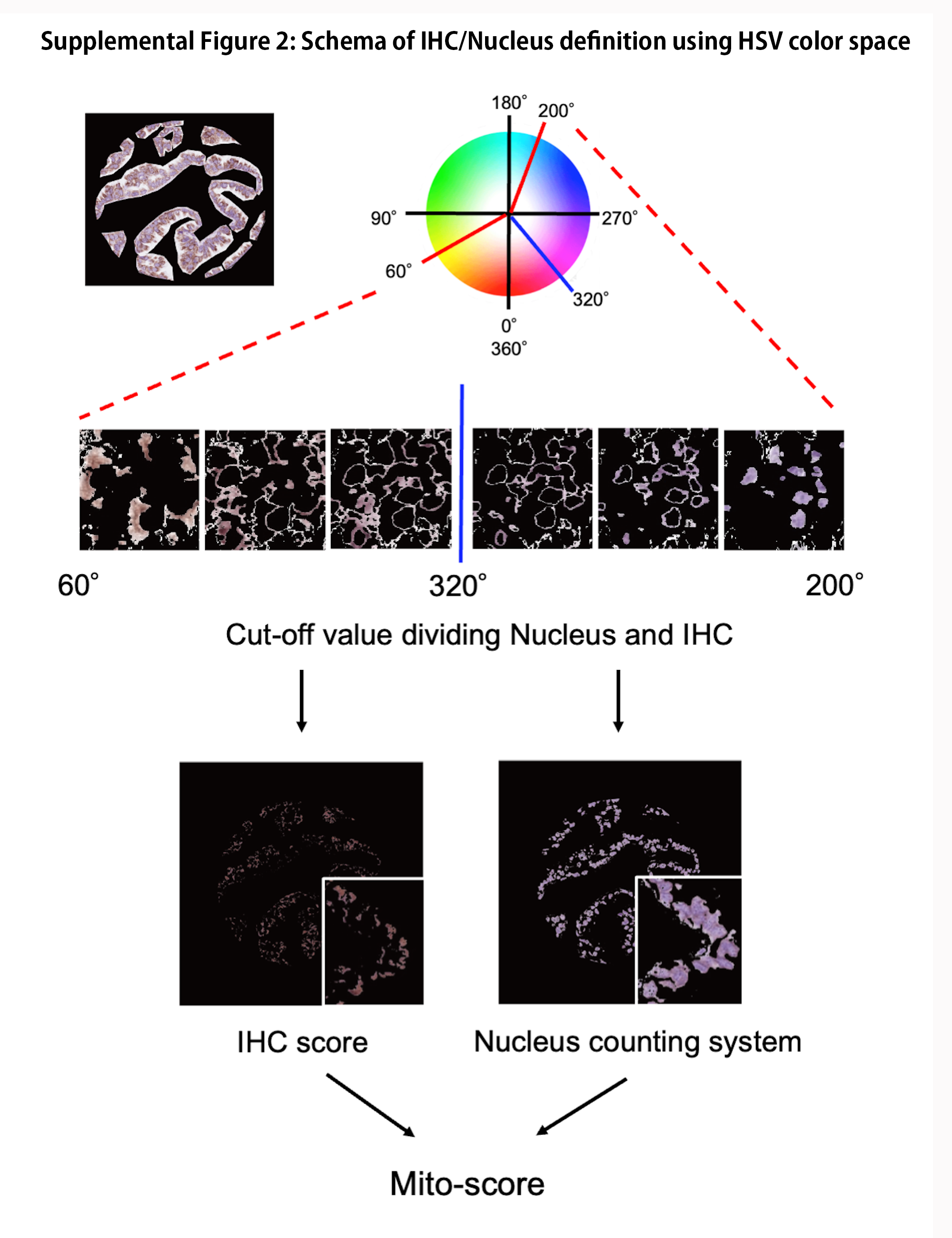

Supplement: Supplementary file 2 — Supplementary file3 (TIFF 17855 kb) [file 10434_2024_16533_MOESM2_ESM.tif]

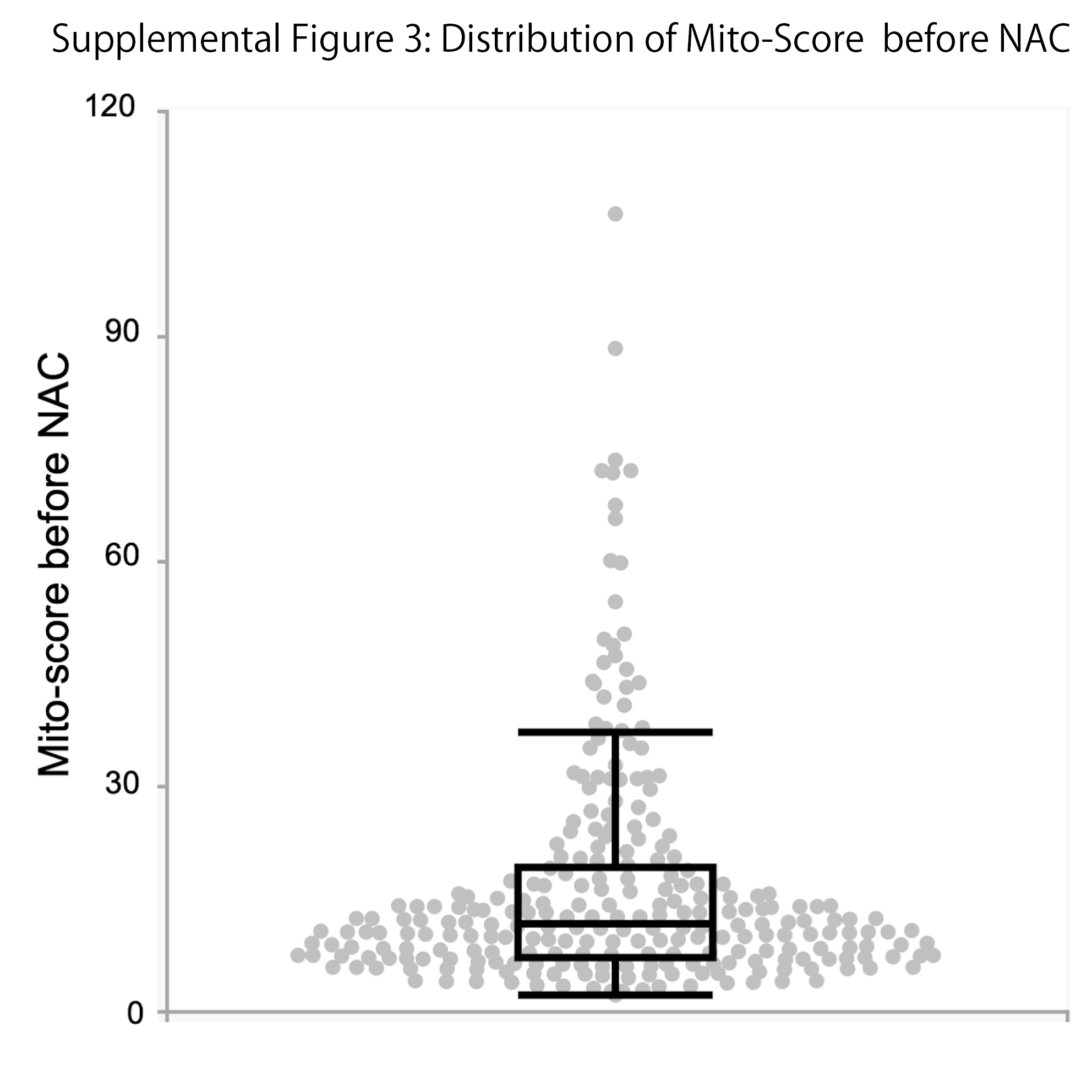

Supplement: Supplementary file 3 — Supplementary file4 (TIFF 11588 kb) [file 10434_2024_16533_MOESM3_ESM.tif]

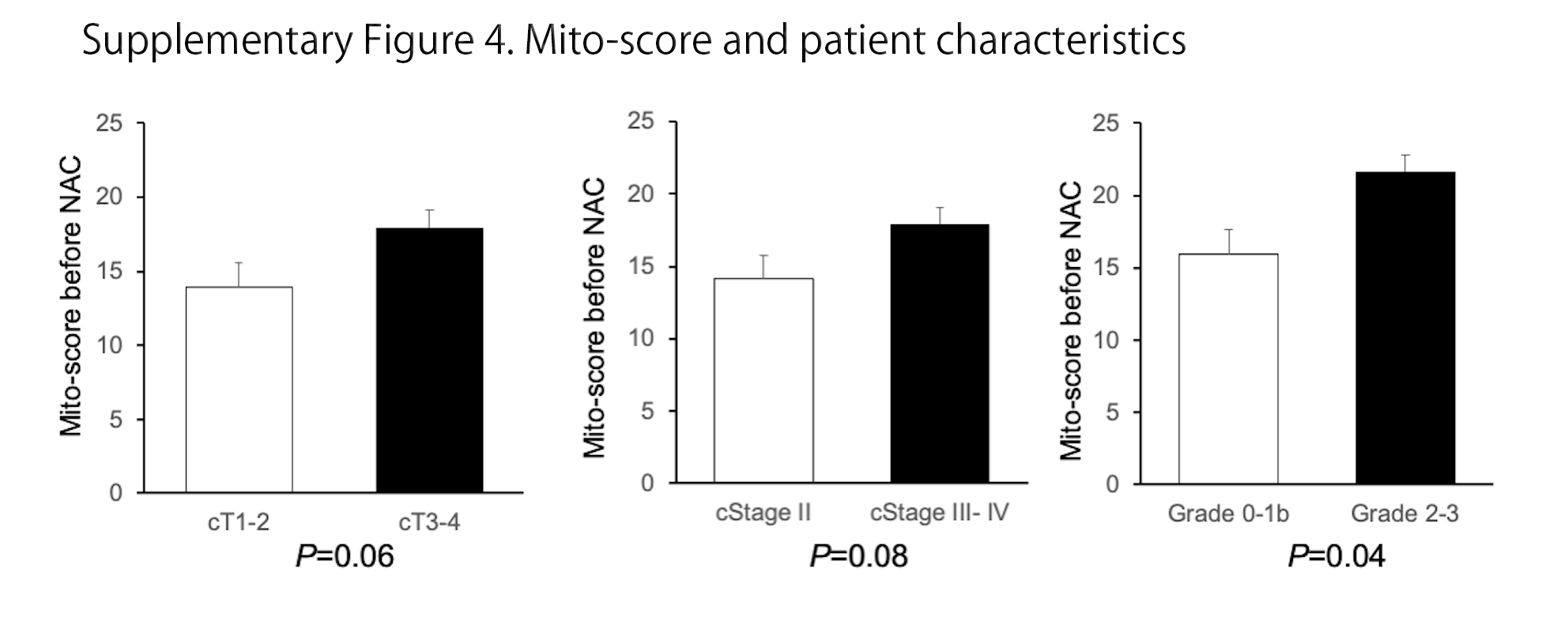

Supplement: Supplementary file 4 — Supplementary file5 (TIFF 4675 kb) [file 10434_2024_16533_MOESM4_ESM.tif]
